# Spatial Variation in Nest‐Site Selection and Population Dynamic of Blue‐Throated Bee‐Eater in a Human‐Altered Landscape: Implications for Conservation

**DOI:** 10.1002/ece3.71828

**Published:** 2025-07-16

**Authors:** Haiying Fan, Weibin Guo, Yanhui Deng, Zuhao Huang, Jiaping Chen, Dianhua Ke

**Affiliations:** ^1^ School of Life Sciences Jinggangshan University Ji'An Jiangxi China; ^2^ Key Laboratory of Jiangxi Province for Biological Invasion and Biosecurity Jinggangshan University Ji'An Jiangxi China; ^3^ Key Laboratory of Jiangxi Province for Functional Biology and Pollution Control in Red Soil Regions Jinggangshan University Ji'An Jiangxi China; ^4^ Library of Jinggangshan University Jinggangshan University Ji'An Jiangxi China

**Keywords:** blue‐throated bee‐eater, habitat fragmentation, long‐term study, nest‐site selection, population dynamic

## Abstract

Anthropogenic activities are among the primary drivers of global biodiversity decline. In conservation practice, monitoring population parameters and clarifying habitat requirements constitute fundamental prerequisites for developing effective strategies. Long‐term research addresses these needs through systematic population monitoring and comprehensive data analysis, establishing critical foundations for biodiversity preservation. This study presents a 15‐year dataset on *Merops viridis* —a nationally protected avian species in China—documenting spatial shifts in nest‐site selection driven by anthropogenic habitat modification and revealing a consistent annual population decline. Our results demonstrate that alterations in nesting habitat critically influence population dynamics, providing theoretical support for evidence‐based conservation strategies. We further discuss potential drivers of observed changes in nest‐site selection and population decline, advocating for the urgent establishment of large‐scale protected areas targeting sandy floodplands to safeguard this species.

## Graphical abstract

Our results indicate that alterations in the nesting site habitat have a crucial impact on population dynamics. This study offers valuable decision‐making foundations for the enhanced conservation of blue‐throated bee‐eaters.

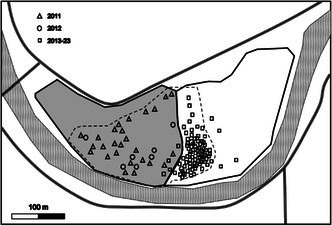

## 1 Introduction

Nest‐site selection represents a critical breeding process in birds by mediating exposure to nest predation (Forstmeier and Weiss 2004), brood parasitism (Zhang et al. 2023), and other factors (Cunningham et al. 2016; Podofillini et al. 2018) that reduce breeding success. Hence, it profoundly influences population dynamics and can even threaten species survival (Clark et al. 2004; Citta and Lindberg 2007), given its pivotal role in determining reproductive outcomes (Chase 2002). This selection often involves specialized preferences for specific habitat characteristics, driven by trade‐offs between competing pressures. For instance, burrow‐nesting offers protection from predators and weather extremes, yet incurs significant excavation costs and microclimate challenges (e.g., gas exchange, thermoregulation). Additional factors like resource proximity, conspecific density, substrate stability, and microhabitat features (e.g., vegetation cover, slope aspect) further shape selection decisions. This specialization is evident in species such as willow tits ( *Poecile montanus* ), which strongly prefer moist habitats with high deciduous tree densities (Vatka et al. 2014), and blue‐tailed bee‐eaters ( *Merops philippinus* ), which selectively excavate burrows in bare sandy loam soils while avoiding vegetation and old nest holes (Yuan et al. 2006).

With the development of society, anthropogenic environmental change is becoming more intense, and one of the most important forms is habitat fragmentation (Palumbi 2001; Fahrig 2003; Xu et al. 2024). Habitat fragmentation poses significant threats to biodiversity, profoundly impacting individual species through mechanisms such as disrupted gene flow, reduced genetic diversity, and increased inbreeding depression (Cartwright et al. 2014; Haddad et al. 2015). Birds are particularly vulnerable to fragmentation effects (Jiang et al. 2008; Xu et al. 2018; Northrup et al. 2019; Perrin et al. 2021) given their extensive global distribution across diverse habitats (Xiao et al. 2017), where even widespread species face population declines when connectivity is lost. However, numerous investigations concentrated on habitat fragmentation effects on species or genetic diversity (Şekercioğlu et al. 2019; Betts et al. 2022; Alemayehu et al. 2024; Mugatha et al. 2024). In order to deeply understand how habitat fragmentation affects nest‐site selection and population dynamics, more longitudinal data based on long‐term studies are needed (Clutton‐Brock and Schldon 2010; Blanc and Thrall 2024). This will improve our understanding of animal population demography and dynamics while providing accurate theoretical guidance for wildlife conservation.

The blue‐throated bee‐eater ( *Merops viridis* ), a migratory species in China, is the most widely distributed member of the Meropidae family in the country. This bird has a gorgeous appearance and is listed as a wildlife species under state protection (del Hoyo et al. 2001). The breeding season in China is from May to September, during which they mainly catch small insects such as bees, butterflies, and dragonflies (Ke et al. 2017). Bee‐eaters have their own special requirements for the soil penetrability of nesting sites. For example, blue‐tailed bee‐eaters ( *M. philippinus* ) typically nest in slopes with soils of sandy loam (Yuan et al. 2006), while European bee‐eaters ( *Merops apiaster* ) primarily occupy loess and sandy hillsides that are covered by vegetation (Kerényi and Ivók 2013; Smalley et al. 2013). The typical nesting habitat of the blue‐throated bee‐eater is flood lands, which are characterized by sandy soil (Ke et al. 2017; Guo et al. 2025). With increasing urbanization, the habitat of nesting sites is gradually changing. However, little is known about the changes in the blue‐throated bee‐eater's nest‐site selection and their impacts on population dynamics in a human‐altered landscape.

During the breeding seasons from 2010 to 2024, we conducted detailed monitoring of the nest‐site selection and population size of a blue‐throated bee‐eater population in Ji'an, Jiangxi Province. Our aim was to provide a empirical basis for the formulation of conservation strategy for this species, via quantifing spatial–temporal changes in nest‐site selection of blue‐throated bee‐eaters in response to anthropogenic habitat modification.

## 2 Materials and Methods

We carried out field work on a flood land at Zhangshan town (27.184° N, 115.048° E), Jizhou district, Ji'an city in the center of Jiangxi Province during the breeding seasons from 2010 to 2024. The annual average air temperature is 19°C and the annual total precipitation is 1504 mm, 80% of which fell between January and August. Summer (June–August) is characterized by high temperatures, frequent rainfall, and contributes substantially to the wet season, based on records from a Ji'an weather station (1980–2016).

**FIGURE 1:**
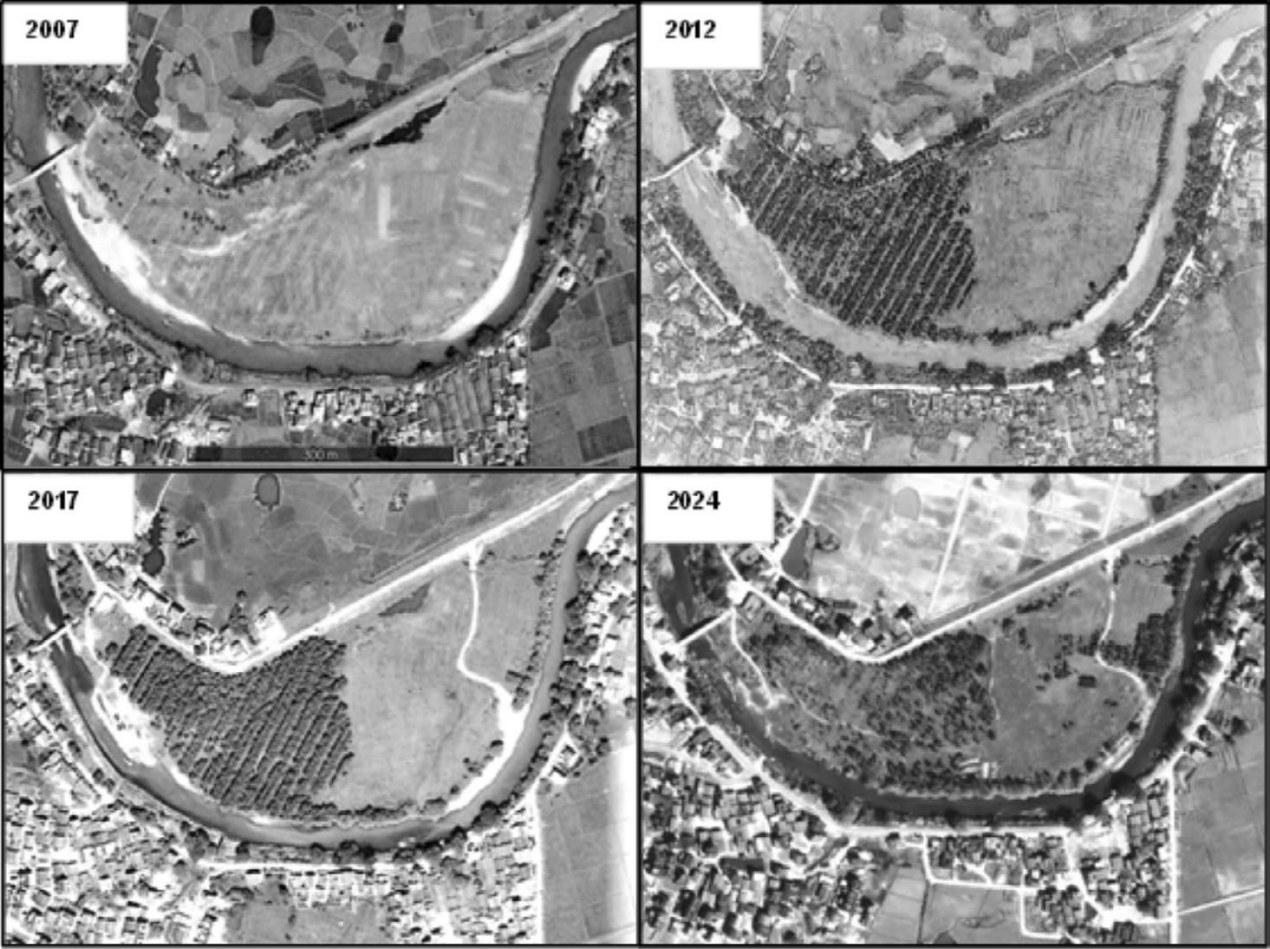
Changes in vegetation coverage across blue‐throated bee‐eater nesting habitat from 2007 to 2024.

Since 2005, half of the area on the west side of the flood land has been planted with pine trees, and the average spacing between pine tree rows is 5 m. According to the satellite map, by 2007, pine trees can be seen in rows. In 2012, there was an obvious gap between the rows of adjacent pines. The canopy arrangement among the ranks of pine trees in 2017 was very tight; in 2020, the pines were partially felled (Figure 1).

We located blue‐throated bee‐eater nests by systematically checking the study area on foot once a week from May to September. Once a nest was found, we determined its precise position using a GPS device and additionally marked the corresponding burrow location on a printed map of the study area. Burrow parameters were measured at different stages: entrance diameter and/or burrow orientation were obtained during the early stages of burrow excavation; burrow length was recorded upon completion of the burrow; and the remaining parameters, such as chamber height, chamber width, chamber length, and soil depth above the chamber, were measured when the burrow was opened to inspect eggs or nestlings. However, during the acquisition of nest data, some records were incomplete. For example, due to the replacement or destruction of nests caused by unavoidable factors, such as agricultural cultivation and urban development activities, only the diameter of the nest entrance was obtained. Annual population sizes were also documented, with the maximum number of adults observed throughout the breeding season instead of the number of breeding pairs being recorded, due to the challenges in reliably identifying paired bonds in dense colonial nesting sites. Clutch size was not incorporated because monitoring this parameter would require invasive nest excavation (e.g., burrow disturbance), which violates ethical guidelines for this protected species and risks altering reproductive behavior. Additionally, rectangular pits (measuring 80 × 60 × 40 cm) were randomly dug in the study area to investigate the correlation between the root growth of the row‐planted pine trees on the flood land and the selection of nest sites. All procedures applied during the research presented were in accordance with the guidelines for animal care outlined by Chinese wildlife conservation laws.

Chi‐square tests were used to examine differences in nest location selection between pine forest and open area habitats across the 2011–2012 and the 2013–2023 breeding seasons, while descriptive statistics characterized burrow orientation for the two periods. Simple linear regression was conducted to assess whether population size (defined as the maximum number of adults) changed during 2012–2024, since late‐season 2011–2012 surveys precluded distinguishing juveniles from adults, yielding only total population counts (including juveniles). The inconsistency in temporal coverage arises because: (1) the population was first discovered late in the 2010 breeding season, allowing only population size documentation that year (no nest sites/structure data were collected), thus nest‐site selection and burrow orientation analyses began in 2011; and (2) breeding sites were cultivated in 2024, resulting in the loss of nest location data, necessitating the exclusion of 2024 from nest‐site selection and burrow orientation analyses. All analyses were performed using SPSS 19.0.

## 3 Results

### 3.1 Nest‐Site Selection and Change in Burrow Orientation

**FIGURE 2:**
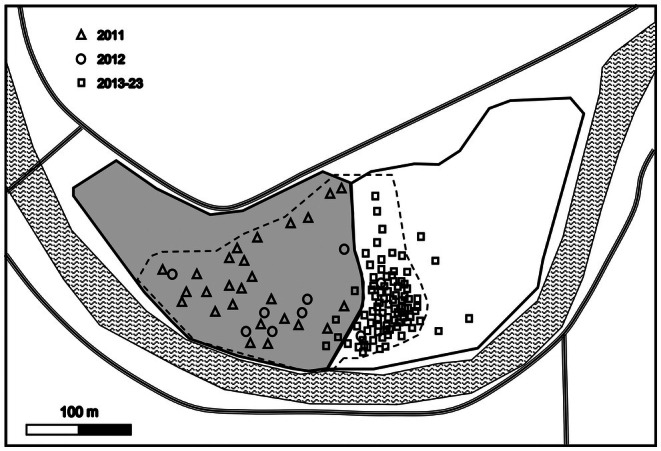
Schematic diagram of the spatial shift in blue‐throated bee‐eater nest site distribution between pine forest and treeless areas during the study period. (Pine forest: Gray background, solid border; Treeless area: White background, solid border; Nest sites: Dashed boxes).

A total of 142 burrows were recorded during the study, and 80.3% (*n* = 114) burrows have tunnel orientation information. Among them, in 2011–2012, burrows were mainly distributed in pine forests (of the 35 nests, 33 were in pine forests, *χ* ^2^ = 27.46, *p* < 0.001). Since 2013, burrows were mainly found in open areas adjacent to the eastern pine forest (of the 79 nests, 75 were in open areas, *χ* ^2^ = 63.81, *p* < 0.001) (Figure 2).

**FIGURE 3:**
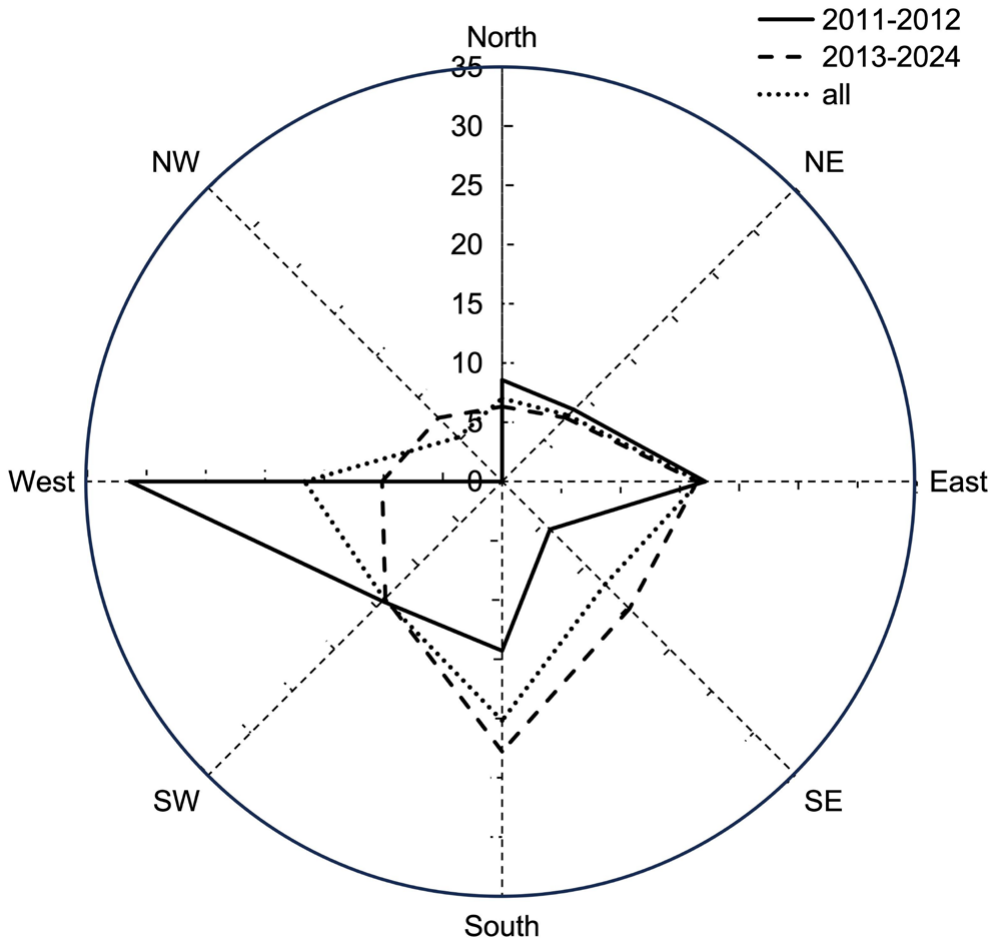
The orientation distribution of blue‐throated bee‐eaters' burrows during the study period. From 2011 to 2012, the nests were mainly distributed in the direction of southwest to northeast (solid line) 2013–2023, the nests were mainly distributed in the direction of south (dotted line).

Accordingly, the orientation of burrows shows significant changes. Among the 35 burrows from 2011 to 2012, nests in pine forest tended to face westerly (17.1%), and less in other directions, such as southeast (5.7%), northward (8.6%), northeast (8.6%), and northwest (0%). From 2013 to 2023, orientations were predominantly distributed in the south (22.8%), east (16.5%), and southeast (15.2%), while less frequent in the north (6.3%), northeast (7.6%), and northwest (7.6%) (Figure 3).

### 3.2 Annual Variation in Population Size

**FIGURE 4:**
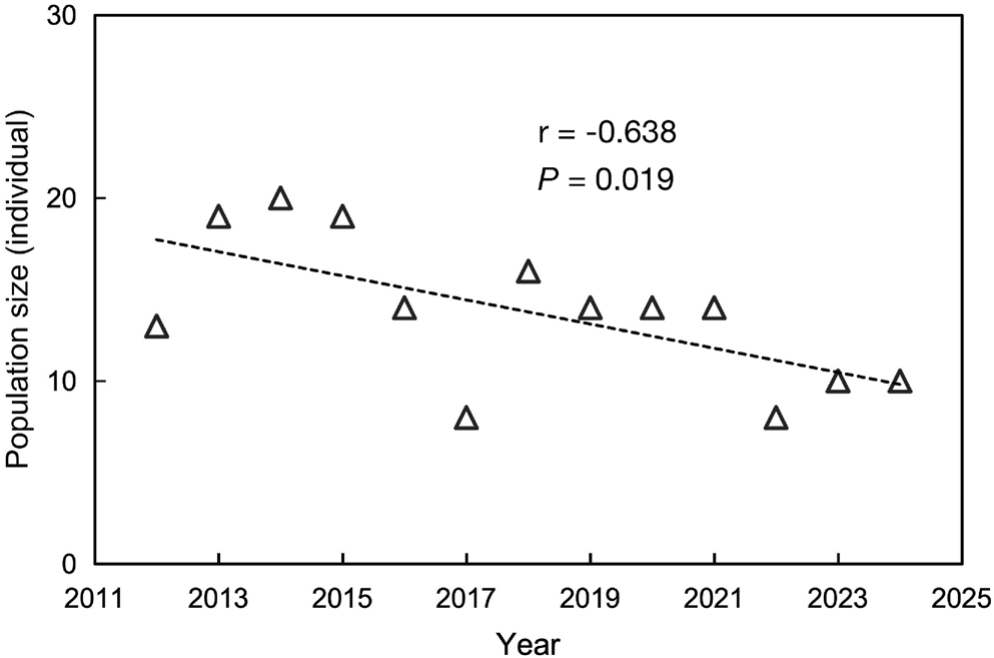
Interannual variation in blue‐throated bee‐eater ( *Merops viridis* ) population size (defined as the maximum number of adults), Zhangshan Town, Ji'an, Jiangxi, 2012–2024.

In 2010 and 2011, the total population (including both adults and juveniles) was 28 and 35 individuals, respectively. From 2012 to 2024, the population size exhibited a significant declining trend (*β* = −0.42, SE = 0.08, *p* < 0.001; Figure 4).

### 3.3 Root Obstruction Documentation

**FIGURE 5:**
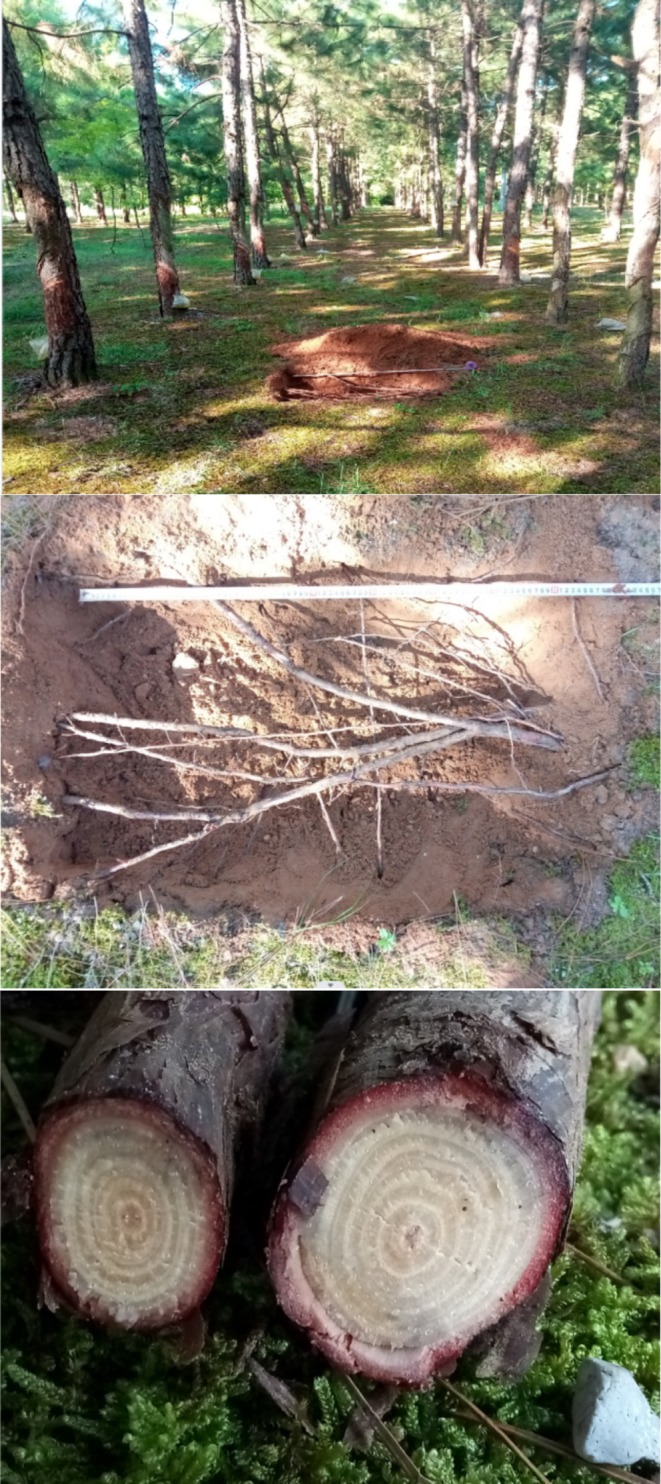
Root obstruction limiting blue‐throated bee‐eater burrow excavation in pine forest sandy soil (2018). (Above: Rectangle pit setting between pine rows; Middle: The pit is covered with pine roots, blocking the extension of the blue‐throated bee‐eaters' burrows. Below: Growth rings of tree roots in the pit).

Physical evidence of root obstruction was documented in 2018, when an attempted burrow excavated midway between two pine rows was discovered filled with lateral roots (Figure 5). Dendrochronological analysis confirmed these roots were 5–6 years old.

## 4 Discussion

Through the 15‐year monitoring of the blue‐throated bee‐eater population in the study area, we documented concurrent spatial shifts in nest‐site selection and a declining population trend. These patterns are driven primarily by anthropogenic habitat modification (e.g., pine plantation development). Rather than driving population dynamics directly, observed changes in nest‐site selection represent a proximate response to deteriorating habitat conditions—particularly reduced availability of suitable excavation zones. The ultimate drivers of population decline are these underlying habitat alterations (e.g., root proliferation in nesting banks), which simultaneously constrain nesting opportunities and reduce carrying capacity. Our results provide a theoretical basis for the formulation of a conservation strategy for this species.

In this study, blue‐throated bee‐eaters excavated burrows in flood land consisting of sandy soil, and the alterations in nest‐site selection and burrow orientation were evidently affected by the presence of planted pine trees. On one hand, the nesting sites shifted from the interior of the pine forest in the early period to the open areas outside the pine forest in the later stage. On the other hand, the burrow orientation in the open area is mainly in the south, east, and southeast, with fewer in other directions. This might be an outcome of adaptive selection in response to ventilation circumstances and thermodynamic impacts (Ke and Lu 2009). However, compared with the open areas outside the pine forest, there were fewer nests within the forest that were perpendicular to the southwest‐northeast alignment of the pine trees. The possible reason is that the horizontal roots impede the blue‐throated bee‐eaters from digging and even lead to the failure of burrowing. Critically, the 5–6 year age of obstructing roots in 2018 coincides temporally with the population's major shift to open areas beginning in 2013. This suggests that the nest‐site selection of blue‐throated bee‐eaters is significantly influenced by the well‐developed underground root systems of pine trees.

Habitat alteration, particularly the loss of suitable nesting sites due to human activities, may be linked to localized population constraints. In the early stage, the nests of the blue‐throated bee‐eaters were mainly distributed within the pine forest. Later, due to the influence of tree roots on nest building, they chose to excavate their nests in the open area outside the pine forest. However, in such open habitats, their nests are frequently damaged as a result of agricultural farming, grazing, and irregular flooding since the open ground is slightly lower in elevation than the pine forest, resulting in low reproductive success and population viability. The same situation also occurred in a comparable sandy flood land approximately 500–1000 m upstream of the study area. In the initial phase, it served as a nesting site for blue‐throated bee‐eaters. Nevertheless, due to agricultural cultivation and development activities, the original environment of the flood land has been severely damaged, leading to the absence of blue‐throated bee‐eaters breeding in this area in subsequent periods. Therefore, we preliminarily infer that the fluctuation in the population size of blue‐throated bee‐eaters may be related to changes in nest‐site selection. Future studies will assess additional factors such as migration, nest predation, and environmental variations to determine the primary drivers of population fluctuations.

Blue‐throated bee‐eaters inhabit ecologically favorable regions where food resources—primarily bees, butterflies, and dragonflies—are typically abundant (Stader 1994; Ke et al. 2017; Huang et al. 2018). Thus, prey availability does not appear to limit population growth under stable conditions. However, during our study period, widespread degradation of nesting habitats across China's breeding range precipitated sharp population declines. Even within designated protected areas (e.g., Qiliping in Hong'an County, Hubei; Huoshan County, Anhui), conservation measures have proven insufficient to counteract habitat loss. Formerly popular observation sites now host diminished populations due to the destruction of critical nesting substrates. This dependence on sandy soils for nesting makes blue‐throated bee‐eaters vulnerable to habitat degradation and underscores the critical importance of conserving these specialized habitats for their population persistence.

Hence, we suggest that any agricultural or other developmental activities within the nesting habitats of the blue‐throated bee‐eater should be strictly prohibited, thereby guaranteeing a stable sandy soil environment conducive to nesting. Meanwhile, the blue‐throated bee‐eaters may need to try multiple locations to succeed in nesting in our study because their tunnels are often blocked by stones or tree roots. Therefore, we recommend constructing artificial dams in blue‐throated bee‐eater breeding areas using fine‐grained sandy substrates with moderate clay/silt content to optimize excavatability while maintaining structural stability. These dams should be engineered to replicate the structural properties of natural nesting banks, featuring vertical to near‐vertical profiles that enable deep tunneling and sufficient compaction to prevent collapse during incubation. Such designed substrates significantly enhance burrow establishment success while providing erosion resistance, thereby supporting population recovery through improved nest‐site availability.

## 5 Conclusion

In conclusion, our results demonstrate that nesting habitat alterations critically influence blue‐throated bee‐eater population dynamics. This study provides valuable decision‐making foundations for enhanced conservation by establishing that protection of frequently overlooked sandy flood plains constitutes the most efficacious measure for population recovery. Nevertheless, several critical knowledge gaps require future investigation to develop comprehensive conservation strategies: the long‐term viability of nesting substrates under climate change, optimal landscape‐scale habitat configuration beyond protected sites, cumulative impacts of hydrological modifications on floodplain integrity, and demographic consequences of alternative management approaches. Addressing these interconnected challenges will be essential for designing holistic conservation strategies that ensure the species' persistent survival.

## Author Contributions

**Haiying Fan:** data curation (lead), formal analysis (equal), funding acquisition (equal), writing – original draft (equal). **Weibin Guo:** data curation (lead), formal analysis (equal), funding acquisition (equal), writing – original draft (equal). **Yanhui Deng:** data curation (supporting). **Zuhao Huang:** data curation (supporting). **Jiaping Chen:** data curation (supporting). **Dianhua Ke:** data curation (lead), formal analysis (equal), funding acquisition (equal), writing – original draft (equal).

## Conflicts of Interest

The authors declare no conflicts of interest.

## Data Availability

This paper does not have additional Supporting Information.
